# Nanoparticle meta-grid for enhanced light extraction from light-emitting devices

**DOI:** 10.1038/s41377-020-00357-w

**Published:** 2020-07-16

**Authors:** Debabrata Sikdar, John B. Pendry, Alexei A. Kornyshev

**Affiliations:** 1 Department of Chemistry, Molecular Sciences Research Hub, Imperial College London, White City, London, W12 0BZ UK; 2 Department of Electronics and Electrical Engineering, Indian Institute of Technology Guwahati, Guwahati, 781039 India; 3 The Blackett Laboratory, Department of Physics, Imperial College London, London, SW7 2AZ UK; 4 Thomas Young Centre for Theory and Simulation of Materials, Imperial College London, London, SW7 2AZ UK

**Keywords:** Applied optics, Lasers, LEDs and light sources, Applied optics, Lasers, LEDs and light sources

## Abstract

Based on a developed theory, we show that introducing a meta-grid of sub-wavelength-sized plasmonic nanoparticles (NPs) into existing semiconductor light-emitting-devices (LEDs) can lead to enhanced transmission of light across the LED-chip/encapsulant interface. This results from destructive interference between light reflected from the chip/encapsulant interface and light reflected by the NP meta-grid, which conspicuously increase the efficiency of light extraction from LEDs. The “meta-grid”, should be inserted on top of a conventional LED chip within its usual encapsulating packaging. As described by the theory, the nanoparticle composition, size, interparticle spacing, and distance from the LED-chip surface can be tailored to facilitate maximal transmission of light emitted from the chip into its encapsulating layer by reducing the Fresnel loss. The analysis shows that transmission across a typical LED-chip/encapsulant interface at the peak emission wavelength can be boosted up to ~99%, which is otherwise mere ~84% at normal incidence. The scheme could provide improved transmission within the photon escape cone over the entire emission spectrum of an LED. This would benefit energy saving, in addition to increasing the lifetime of LEDs by reducing heating. Potentially, the scheme will be easy to implement and adopt into existing semiconductor-device technologies, and it can be used separately or in conjunction with other methods for mitigating the critical angle loss in LEDs.

## Tailored meta-grid of nanoparticles could boost performance of light-emitting diodes

Introducing a tailored layer of plasmonic nanoparticles into the epoxy casing of an LED can improve the device’s light output, which would benefit energy savings and boost LED lifetime. D.S. from IIT Guwahati along with J.P. and A.A.K. from Imperial College London revealed the benefits of adding a two-dimensional array of silver nanoparticles called a “meta-grid” to the lens-shaped epoxy packaging. Their theory, tested by computer simulations, specifies optimal conditions for the nanoparticles’ size, composition, spacing, and location, for which such meta-grids significantly reduce the Fresnel reflection loss at the LED-chip/epoxy interface, thus improving light extraction from LED. The approach is customizable to suit a specific emission color or the entire emission spectrum of a typical broadband LED. Authors proposed a few schemes to implement it into the existing LED manufacturing technology.

## Introduction

Light-emitting diodes (LEDs) are overwhelmingly used in the modern world—from traffic lights to backlighting for electronic displays large outdoor screens, and general lighting/decorations and in sensing, water purification, and decontamination^1,2^. There have been numerous research attempts over the years to further enhance the light extraction efficiency of LEDs, toward producing greater light output^2–12^. The efficiency of light extraction from conventional semiconductor LEDs [where an LED-chip of refractive index *n*_1_ (between 2.4 and 3.6) is encapsulated by a transparent insulator of refractive index *n*_2_ (between 1.4 and 1.6)] is limited mainly by two inherent issues^2,12^.

First, as the chip-encapsulating material (e.g., epoxy, plastic, or glass) has a much lower refractive index than the chip, the amount of light extracted from the chip into the encapsulating casing is reduced due to the restrictions imposed by the critical angle calculated from the normal to the chip/encapsulant interface^13,14^, *θ*_c_ = arcsin (*n*_2_/*n*_1_).When light impinges on the interface at such an angle, the refracted light escapes along the interface. For angles larger than *θ*_c_, the incident light undergoes total-internal reflection and no light escapes into the encapsulating casing, accounting for the critical angle loss.

Second, even at angles smaller than *θ*_c_ i.e., within the photon escape cone, a significant fraction of the incident light is reflected back from the interface into the chip—corresponding to the Fresnel loss^2,13,14^. The larger the difference between *n*_1_ and *n*_2_ is, the greater the loss. For example, in a typical semiconductor LED chip (*n*_1_ = 3.5) with an encapsulating casing (*n*_2_ = 1.6) only 84% of the generated light that impinges normally onto the chip/casing interface can escape into the casing. Therefore, increasing transmission across the chip/encapsulant interface would increase the overall light extraction from LEDs.

To mitigate these issues, previously, the use of chalcogenide glasses with higher refractive indices than epoxy/plastic has been proposed^2,12^. However, this idea faces significant difficulties in adaptation to mass production of LEDs as different equipment is needed in processing these glasses, which are also typically not very transparent. A proposal for creating LED chips of a hemispherical shape to reduce total-internal reflection by ensuring that the incident angle is always less than *θ*_c_, thus minimizing the critical angle loss^2^, was also presented. However, hemispherical LED chips are bulky, and their fabrication is more difficult and uneconomical for mass production^2^. There are other interesting proposals including coating LEDs with a spatially-gradient refractive index material to help reduce the Fresnel loss; in addition, coating LEDs with a SiO2/PS microlens array could help increase the photon escape cone to reduce the critical angle loss^15^.

Many further schemes to increase the effective refractive index of the packaging material that all can improve the light extraction efficiency have been proposed and tested^16–21^ using either nanoparticle-epoxy nanocomposite or nanoparticle-loaded-epoxy or epoxy materials incorporated with filler powders or engineered epoxy resins to name a few. The purpose of populating the encapsulating material with nanoparticles (NPs)—composed of transparent metal oxides (or combination of metal oxides, a group II–VI materials, or alloys of Zn, Se, S, Te, Ti, and Zr, or even GaN, SiN, or AlN) with sub-wavelength sizes ranging from 1/5 to 1/20 of the LED wavelength—all of larger refractive index than of the host, is as follows. The NP density should ensure a higher refractive index of the composite without compromising its transparency. However, controlling the density and arrangement of NPs in the host without agglomeration while preserving the transparency, in addition to mitigating adverse effects from a large NP sides dispersion on LEDs of different emission wavelengths, is difficult. Moreover, a larger refractive index of the encapsulant could lead to a larger portion of light being reflected back from the encapsulant/air interface.

This paper proposes an alternative route for improving light extraction from LEDs. It suggests increasing the light transmission across the chip/encapsulant interface by reducing the Fresnel loss at the chip/encapsulant interface within a fixed photon escape cone, while prescribing minimal changes to the manufacturing process. The scheme can be used in isolation or in conjunction with other methods for mitigating critical angle losses. The proposed idea is to place a monolayer of sub-wavelength metallic NPs, acting as “meta-grid”, on top of a conventional LED chip within the chip’s usual encapsulating packaging. We will show that within an optimized range of the NP meta-grid parameters, such modification of LED structure could significantly boost the LED light transmission across the chip/encapsulant interface, within the photon escape cone, for any spectral range of emission. The enhancement is based on destructive interference between light reflected from the chip/epoxy interface and light reflected by the NP meta-grid. Reducing reflection from the chip/epoxy interface can also increase the lifetime of the LED chip by eliminating heating of the chip from unwanted reflections within the chip.

**Fig. 1:**
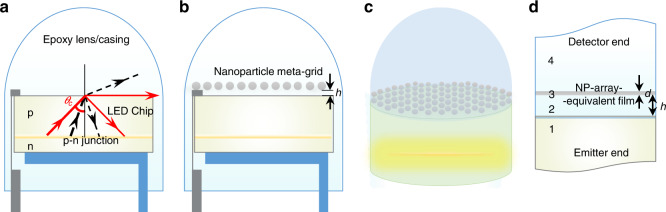
Schematics of the classical and modified light-emitting diode (LED) device (not to scale). **a** Cross-sectional cartoon of a standard LED (including its electrical contacts), where an epoxy lens/casing encapsulates the semiconductor LED chip. Light emitted from the p–n junction escapes into the epoxy lens as long as the incident angle is less than the critical angle *θ*_c_. **b** Side-view and **c** 3D view of the proposed new design for enhanced light extraction with a 2D array (“meta-grid”) of nanoparticles (NPs) embedded in the epoxy material at a height *h* from the LED-chip surface. **d** Four-layer-stack theoretical model for analysing the optical transmission through the proposed system, where the NP array is represented by an effective film of thickness *d*, whose properties are derived from the effective medium theory (see section: “Theoretical Formulation”)

Figure 1a depicts a cross-sectional view of a typical semiconductor LED, in which a hemispherical lens/casing of an insulating packaging material (e.g., epoxy, plastic, or glass) encapsulates an LED chip. At an incident angle of *θ*_c_, light emitted from the chip’s p–n junction escapes only along the chip/encapsulant interface, with some portion being reflected back (red arrows). Generated light can only escape into the epoxy casing if the incident angle at the interface is less than the critical angle *θ*_c_, i.e., within the photon escape cone of the interface (black dashed arrows). However, there is still unwanted reflection from the interface in these cases. We focus on reducing these reflections within the photon escape cone of the device, while extracting the generated light.

Our proposed design deploys a monolayer of sub-wavelength sized metallic NPs acting as a “meta-grid” on top of the conventional LED-chip embedded at a height *h*, within the chip’s standard encapsulating packaging (see Fig. 1b, c). Figure 1d depicts a four-layer-stack model for analyzing the optical transmission through the proposed system, the details of which are presented below.

## Results

For a demonstration of our concept of NP-assisted enhanced transmission, we consider silver nanospheres. Silver makes the strongest plasmonic resonator with minimal absorption losses^22–25^, making it an obvious choice. First, we investigate the roles of NP radius *R*, interparticle gap *g* in the 2D hexagonal array, and “height” *h*. Note that, if the NP meta-grid is fabricated from a bottom-up self-assembly process, then it is usually the case that the nanospheres will arrange themselves in a 2D hexagonal array. If one aims at fabricating the meta-grid in top-down approach, then again hexagonal array would be preferred over square or other lattices, as hexagonal lattice has comparatively higher packing density which could produce the strongest effect from an NP meta-grid.

**Fig. 2:**
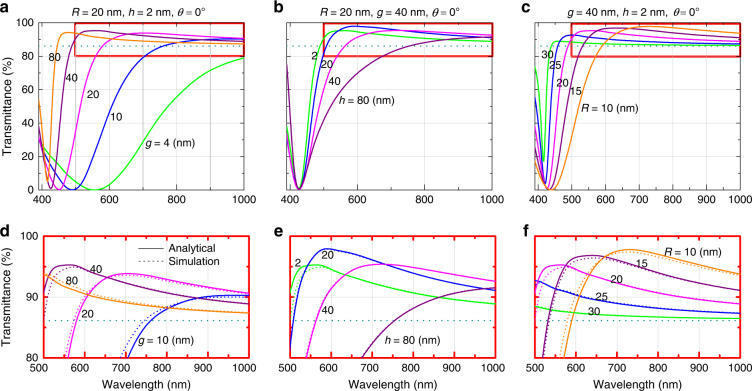
Transmission spectra depicting effects of different physical paramaters of NP meta-grid. Transmittance spectra, calculated from the theoretical model, depicting the effects of different physical parameters of the hexagonal array of silver nanospheres, such as the radius *R*, interparticle gap *g*, and “height” *h* from the interface between typical semiconductor (*n*_1_=3.5) and encapsulating (*n*_2_=1.6) materials: **a** variation with *g* for fixed radius (*R*=20nm) and height (*h*=2nm), **b** variation with *h* for fixed radius (*R*=20nm) and gap (*g*=40nm), and **c** variation with *R* for fixed gap (*g*=40nm) and height (*h*=2nm). **d**–**f** Zoomed-in view of the theory-based (“analytical”) spectra, in the domains highlighted by the red boxes in (**a**–**c**) compared with data (colored dotted curves) obtained from full-wave simulations. For all cases, only normally incident light is considered. The dotted horizontal lines indicate transmittance without the nanoparticle layer

Examples of the theoretically calculated transmittance spectra at normal incidence of light for different values of these parameters are depicted in Fig. 2. Note that throughout this paper, the transmittance is calculated with a light emitter and a detector placed inside the chip and the encapsulating medium, respectively (see Fig. 1d). The permittivity of silver is taken from the literature^26^. The dotted horizontal lines in all cases show the transmittance in the absence of the NP layer. It is seen that different sets of parameters of the NP array provide the maximum enhancement in transmission over different spectral windows. Therefore, the design of the NP “meta-grid” should be optimized for each LED depending on its emission spectral range.

Figure 2d–f show the transmittance spectra highlighted in the red boxes in Fig. 2a–c, respectively, along with the colored dotted curves obtained from full-wave simulations. The close correspondence between the analytical and simulation spectra indicates that our analytical approach is quite accurate. Hence, our theoretical model can be safely deployed for finding the optimal design of the NP meta-grid for any specific LED application.

Note that, the transmitted light through an NP meta-grid is essentially that portion of the incident light which remains after getting extinct (absorbed plus reflected) from its path by the NPs. Therefore, to understand the fundamental mechanism behind the changes in the transmittance spectrum with variation of the physical parameters of an NP meta-grid, as shown in Fig. 2, one needs to think of the effects of these parameters on the corresponding extinction spectra. Optical response of an NP meta-grid usually features an extinction peak, associated with excitation of localized plasmon resonances in NPs interacting with each other in the array. This extinction’s peak position and width depend on the meta-grid parameters.

This extinction peak appears as a *dip* in the corresponding transmittance spectrum. The narrower and shallower is the transmission dip, the higher is the overall transmittance averaged over a specified wavelength window. In each transmittance spectrum, there is a steep rise next to the dip (on its longer-wavelength side) with a maximum, featuring a decaying tail extending towards red. That part of transmittance spectrum plays a vital role in the average transmittance over a spectral range.

**Fig. 3:**
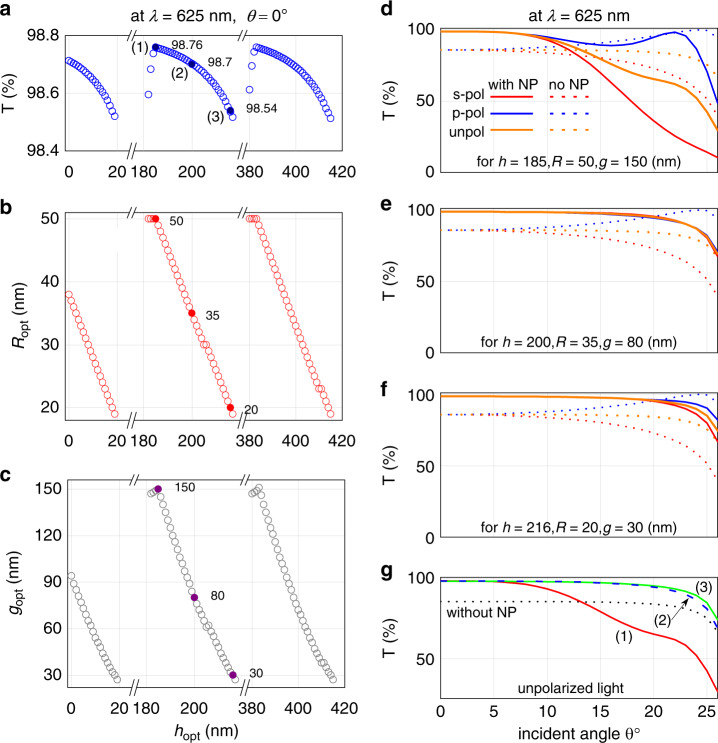
Obtaining parameters for optimal transmission and its dependence on incident angle. **a**–**c** Optimization of the optical transmittance (*T*) at 625nm for normal incidence via tuning of the NP array parameters. **a** Maximum transmittance obtained at each height *h* (where *T*≥98.5%), and corresponding optimal (**b**) radius *R*_opt_, and (**c**) interparticle gap *g*_opt_. **d**–**f** Transmittance at different permissible incident angles for s-polarized (red), p-polarized (blue), and unpolarized (green) light for cases (1)–(3) [marked in (**a**)]; for each polarization, the dotted curves show the light transmission without the NP array. **g** Comparison among the transmittance for unpolarized light in those three cases. The dotted line, obtained without the NP array, serves as a reference. Here, AlGaInP (*n*_1_=3.49) is the semiconductor material and epoxy (*n*_2_=1.58) is the encapsulating material

In this paper, our objective is to maximize transmittance over a specified spectral range using an optimized structure of the meta-grid. In an ideal scenario, one has to choose meta-grid parameters in such a way that an extinction peak (or the transmittance dip) falls outside the spectral range of LED emission. In Fig. 2, with yet no specific LED spectral window in mind, we describe that the effect of NP meta-grid’s parameters on transmittance spectrum in general. Hence, these spectra are depicted over a much wider range, from 400 to 1000 nm. The enhancement in transmission with the NP meta-grid, as compared with the case when no meta-grid is present, can be attributed to the Fabry–Perot effect between chip/encapsulant interface and NP meta-grid, as discussed in detail later with Fig. 3. Here, we discuss parametric dependence of the transmission dip (extinction peak) to understand the physics behind it.

With reduction of inter-NP gap *g*, at other parameters of an NP meta-grid unchanged, interparticle coupling within the meta-grid gets stronger, and the transmission dip (or the extinction peak) gets redshifted and broader^27^. This is, however, not desirable in our case. Broadening of a transmission dip and moving its minimum to the center of the targeted spectral range will result in lowering of the average transmittance. This can be seen in Fig. 2a, where the average transmittance for *g* = 4 nm over the depicted spectral window is significantly lower due to a broad transmittance dip as compared with the other considered cases. With increase in *g*, transmittance dip moves farther to shorter wavelength; but the maximum of transmittance also moves together with the dip, and the tail on the longer-wavelength side of the maximum decays faster, and therefore such sparse array does not provide the maximal average transmittance in the depicted spectral range. The reason why the tail decays faster for larger *g* is because the plasmonic coupling among NPs weakens at a steeper rate for larger *g*.

With increase in height *h* of the NP meta-grid from the chip/encapsulant interface, there is no significant change in the location of the transmittance dip (extinction peak) (see Fig. 2b), as long as the NP meta-grid’s composition remains unchanged. There is, however, some spectral broadening in the transmission dip with increase in *h*, which may be attributed to weakening of the extinction resonance peak due to reduction in the image-charge interactions on the NP meta-grid^27^. This broadening of transmission dip is not desirable in our application scenario.

Furthermore, with increase in radius *R* of the silver NPs at a fixed *g* and *h*, the transmittance dip gets blue-shifted, weaker, and narrower (see Fig. 2c). This can be attributed to the reduction in the image-charge interactions with increase in *R*^27–29^. Although this trend looks beneficial toward achieving higher average transmittance, larger absorption by the added “plasmonic mass” of the NPs attenuates transmission over a wide spectral range and the benefit of transmission enhancement by NP meta-grid gradually disappears with further increase in NP size.

Now, we consider a specific case of a typical red LED with a peak emission wavelength of 625 nm, where AlGaInP (*n*_1_ = 3.49) is the semiconductor material and epoxy (*n*_2_ = 1.58) is the encapsulating material. To determine the parameters for the optimized configuration of the NP meta-grid, the study considered the following range of parameters: *h* from 0 to 500 nm, *R* from 5 to 50 nm, and *g* from 1 to 250 nm, all varied at steps of 1 nm.

Figures 3a–c depict the transmittance (*T*) optimized at each height h (provided the optimized *T* ≥ 98.5%) at normal incidence. For all these cases, the optimal transmittance value, its corresponding optimal radius *R*_opt_ and its corresponding optimal gap *g*_opt_ are plotted in Fig. 3a–c, respectively. Note that the transmission level obtained at any *h* repeats for other heights of *h* + *m* × λ/(4 × *n*_2_), with *m* being a positive integer. This suggests that the Fabry–Perot effect is behind the transmission enhancement—where light reflected from the chip/encapsulant interface destructively interferes with the light reflected by the NP array to effectively reduce reflection from, (in other words, increase the transmission through) the chip/encapsulant interface. Fabry–Perot effects can be seen between any two parallel reflective surfaces, however, the higher is the reflectivity of the reflective surfaces the better is the quality factor of the resonance peak. We have shown Fabry–Perot cavity effects in our previous works^30,31^ between two parallel NP-layers, with moderate or high reflectivity. In this work, the chip/encapsulant interface and the NP meta-grid act as the two reflective surfaces forming the cavity in between them, where encapsulating material acts as the cavity medium. Self-absorption in the semiconductor emissive layer will not alter the property of the cavity, other than the fact that with stronger self-absorption lesser amount of light will be able to escape the chip/encapsulant interface. It is, therefore, theoretically possible to position the NP array at any of those heights *h*, where the transmission enhancement conditions are met according to the Fabry–Perot cavity theory. However, it would be wiser in practice to place the meta-grid at the closest possible height to the chip/encapsulant interface, in order to restrict any leakage of radiation from the sides before impinging on the meta-grid.

Note that Figs. 2, 3a depict transmittance spectra at normal incidence where s- and p-polarized light are transmitted in the same way. Figure 3a also marks three distinct points are marked as (1), (2), and (3), for which *R*_opt_ and *g*_opt_ are shown as the filled circles with values listed in Fig. 3b, c respectively. These cases are further investigated for all permissible incident angles below the critical angle. For off-normal incidence, s- and p-polarized light are transmitted differently. Hence, transmittance for the case of unpolarized light, which is typically the state of the light emitted from the emissive layer, is obtained by averaging transmittances of s- and p-polarized light at any incident angle.

Figure 3d–f depict the transmittance for s-polarized (red), p-polarized (blue), and unpolarized (green) light for cases (1)–(3), respectively, at different incident angles with/without the NP array. Case (1), although it provides the maximum *T* at normal incidence, is strongly polarization sensitive at off-normal incidence angles (the manifestation of this effect becomes stronger with increasing NP size) (Fig. 3d). For smaller NPs, in cases (2) and (3), polarization effect becomes negligible, for all permissible angles (Fig. 3e, f). For unpolarized light, such as the light emitted from the LED chip, case (3) shows the largest transmittance over all permissible angles with the best angle-averaged transmittance. A comparison among the three cases shows that small NPs could exhibit better angle-averaged transmittance for unpolarized light (Fig. 3g). Clearly, the optimization procedure must consider the transmittance averaged over all allowed angles within the photon escape cone, not just that at normal incidence.

Another important aspect to consider is the fact that the typical emission spectrum of any commercial LED (of any color) has a finite spectral width. For example, the emission spectrum of a typical AlGaInP/GaAs red LED by Toyoda Gosei Corp. ranges from 580 nm to 700 nm^2^. Therefore, we conduct the next optimization study over the abovementioned s spectral range and try maximize the average transmittance over all permissible angles. The range can be customized according to the specific emission spectrum of an existing LED chip on top of which the NP “meta-grid” will be positioned.

**Fig. 4:**
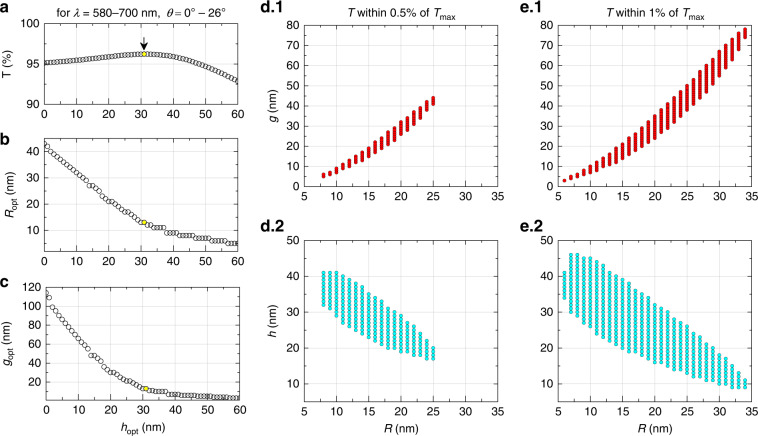
Finding meta-grid parameters for optimal transmittance of a broadband LED. **a**–**c** Optimal transmittance and corresponding nanoparticle array parameters obtained for various heights (distances) of the NP array from the LED-chip surface. The maximal transmittance *T* (**a**) achievable for different heights *h*, and the corresponding optimal radius (**b**) and corresponding optimal gap (**c**). In (**a**), the data for *T* represent transmittance averaged over a spectral range of 580–700nm and over incident angles ranging between 0° and 26°, at specific *h*. The yellow dots represent the optimum point of transmission *T*_max_=96.2%, obtained at an optimal (**a**) height *h*_opt_=33nm; and (**b**) optimal NP meta-grid parameters: (**b**) *R*_opt_=13nm and (**c**) *g*_opt_=13nm. **d**–**e** Optimizing gap and height for different values of particle radii that allow transmission within 0.5% (**d**) and within 1% (**e**) of *T*_max_. Note that calculations within the spectral window between 580 and 700nm were performed at a step of 1nm, and angles between 0° and 26° were taken at a step of 1°. Beyond the critical angle (~26°) light emitted from the LED-chip undergoes total-internal reflection, thus failing to escape into the epoxy lens. Here, AlGaInP (*n*=3.49) is the semiconductor material and epoxy (*n*=1.58) is the encapsulating material

Figure 4a–c depict the optimal transmittance (*T*) and corresponding NP array parameters for different heights (*h*). The displayed range is from 0 to 60 nm, beyond which the transmittance gradually decreases and is hence insignificant. The transmittance described here is calculated by averaging over a spectral range of 580–700 nm and over incident angles ranging between 0° and 26°. The yellow dots represent the optimum point of the maximum transmittance (*T*_max_ = 96.2%) at *h*_opt_ = 33 nm, *R*_opt_ = 13 nm, and *g*_opt_ = 13 nm. This enhanced transmittance, achieved in the presence of the optimized NP meta-grid, is significantly larger than the otherwise obtained transmittance of 83.9%, without any NPs, over the same range of wavelengths and incident angles. The study reveals a trend in which both the optimal radius and interparticle gap decrease with height. We also show all combinations of the parameters of the NP array (interparticle gap and height for specified NP radii) that can provide transmission within 0.5% and 1% of *T*_max_ in Fig. 4d, e, respectively.

**Fig. 5:**
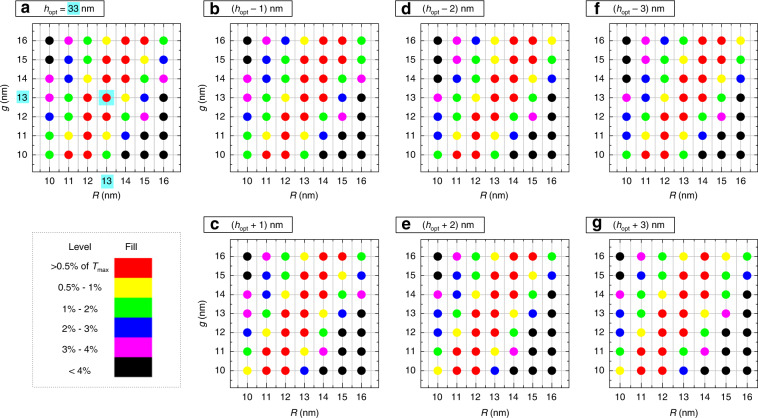
Optimization of the transmittance (over a spectral window of 580–700 nm averaged over all permitted incident angles (below the critical angle) and its sensitivity to the NP meta-grid parameters. **a** Dots with different fill colors depicting the deviation from the maximum transmittance (*T*_max_) for a fixed height of *h*_opt_ = 33 nm but various radius *R* and gap *g*, where both these parameters are assumed to be larger/smaller than their optimal values by up to 3 nm. *T*_max_ (of 96.2%) is achieved at the optimal height *h*_opt_ = 33 nm, for the optimal radius of 13 nm and gap of 13 nm [highlighted in cyan]. **b**–**g** Same as in (**a**), but for different heights of (*h*_opt_ − 1), (*h*_opt_ + 1), (*h*_opt_ − 2), (*h*_opt_ + 2), (*h*_opt_ − 3), (*h*_opt_ + 3), respectively. Note that, for the calculations the spectral window between 580 and 700 nm was considered at a step of 1 nm and angles between 0° and 26° were taken at a step of 1°. Here, AlGaInP (*n* = 3.49) is the semiconductor material and epoxy (*n* = 1.58) is the encapsulating material

It is also interesting to see how sensitive the maximum transmittance level is to any imperfections in the fabrication process, leading to deviations from the optimal values for the NP radius and inter-NP gap. Figure 5a plots the transmittance levels achieved for all combinations of *R*_opt_ ± 3 nm and *g*_opt_ ± 3 nm at the optimal height *h*_opt_ = 33 nm. Note that the figure is a 2D map of transmittance levels in the *R*–*g* plane with (*R*_opt_, *g*_opt_) at the center (highlighted in cyan) for a fixed height of *h*_opt_—where the dots filled with different colors represent different levels of percentage reduction of transmission from *T*_max_. Similar 2D maps of transmittance are also plotted for different *h* values ranging from *h*_opt_ − 3 nm to *h*_opt_ + 3 nm (Fig. 5b–g). In Figs. 4, 5, the transmittance data correspond to unpolarized state of light emitted from the emissive layer.

Knowing the optimal configuration of the NP array, the next step is to prepare silver nanospheres of radius *R*_opt_, coated/functionalized with ligands of appropriate length to ensure that an interparticle gap of *g*_opt_ is obtained. A monolayer of these NPs can be fabricated using the drying-mediated self-assembly method, as done for “plasmene”^22,23^. The NP meta-grid can be prepared on a substrate (ideally of the same material as the encapsulant), where the substrate thickness is chosen to correspond to *h*_opt_. In another approach, first, the encapsulating layer of height *h*_opt_ can be deposited on the LED chip, and then, the NP monolayer can be fabricated on or transferred on to it^22,23^. Such a meta-grid of NPs formed on a substrate (a stretchable substrate would also allow for precise tuning or further adjustment of the interparticle gap) could then be stamped^23,32,33^ onto the LED chip. After the NP layer is deposited, the encapsulant material with can be spin coated on it. This would ensure that the embedding medium of the NP monolayer is the same as the encapsulant and after that, the usual hemispherical casing can be fabricated or inserted.

## Discussion

The various aspects of our proposed scheme are summarized below:

A significant enhancement in light extraction from LEDs can be achieved by boosting the transmission across chip/encapsulant interface, by introducing a monolayer of plasmonic NPs on top of the LED chip which can reduce the Fresnel reflection loss at the chip/encapsulant interface, through enhanced transmission originating from the Fabry–Perot effect. A similar effect is also applicable for enhancing the trapping of light in solar cells^34^.

This scheme can be deployed by itself or in combination with other schemes available for increasing the LED efficiency by reducing critical angle losses.

The theoretical model allows determination of the optimal conditions for the structure and properties of the NP layer: viz. the material and composition of NPs, their sizes and average interparticle spacing, and the distance from the surface of the LED chip—that could provide the maximum enhancement in light extraction from the LED chip into the encapsulating casing, over the LED emission spectral range. It must also be noted that although we have demonstrated our proof-of-the-concept using hexagonal lattice arrangement of NPs, the idea and the physics of transmission enhancement using NP meta-grid will remain unchanged for any other lattice arrangement of NPs in a meta-grid. In the effective medium theory with which we described the system, changing the structure of the grid will affect only the lattice sums [Eq. (3), “Materials and methods”]; these will need to be recalculated, and the optimization study will need redoing to find out the best values parameters for the meta-grid parameters. In the COMSOL finite-element-method (FEM) simulations, the starting unit cell is to be assigned a different structure. But it is obvious that for any densely packed structures the effect will not be qualitatively different, whereas the hexagonal structure will let to achieve the maximal possible light extraction.

With continuous advancement in nanofabrication technology, it is becoming less difficult to fabricate the NPs which are mostly monodisperse and have a very narrow spread. Still, there could always be some randomness in particle size and/or position, flatness of grid, and variation in refractive index due to fabrication error or material defects, which are unavoidable. Effects from most of these inaccuracies can be roughly estimated from our tolerance study. This is what Figs. 4, 5 present and we discussed it in detail. With such fabrication errors, we may not get the optimal *T*_max_, but in most cases, we will still be able to achieve transmittance *T* within 0.5% or 1% of *T*_max_, which will still be a significant boost in light transmission across chip/encapsulant interface. This is the message we wanted to convey with our tolerance study. It may not include all sorts of inaccuracies, but definitely shows a way to handle these issues while optimizing the meta-grid design and choose optimal parameters for in a particular design specific to a type of LED.

The design and operation of the NP meta-grid for the enhanced light transmission/extraction scheme presented here are substantiated by the originally developed theory, verified against standard full-wave simulations.

Using this theoretical framework, the optimal design of the NP meta-grid can be tailored according to the materials used for the LED chip and encapsulant, operating over any spectral window.

One method for realizing the NP meta-grid is to use drying-mediated self-assembly of NPs (e.g., NPs made of silver or alternative less-lossy plasmonic materials capped with appropriate ligands) to form free-standing “plasmene” sheets. The NP monolayer could also be conformed to a stretchable substrate (of the same encapsulating material) for precise tuning of the interparticle separation and could be stamped on the LED chip before the encapsulating casing is fabricated/inserted. The distance of the NP array from the LED chip surface can be controlled via the thickness of the plasmene’s substrate.

The simplicity of the proposed scheme would make it easily adaptable to the existing LED manufacturing process. It is obvious that with larger light extraction efficiency, LEDs will provide greater energy savings as well as longer lifetimes of the devices, which will have a global impact on diverse LED based applications. In the presented study, we have demonstrated the effect of NP meta-grid for the standard commercial LEDs, based on III–V group materials. But the proposed concept of enhancing light transmission from an emissive layer to its encapsulant casing may also be applicable to other types of light-emitting devices containing emissive-layer/encapsulant interface. Generally, our idea of using the NP meta-grid for enhanced light extraction could potentially cater to a wider range of optical gadgets, not just semiconductor LEDs, although this class of devices is our primary focus in this paper.

Our paper primarily focuses on improving the light extraction across LED chip/encapsulant interface by reducing the Fresnel loss, which will definitely improve the whole device performance. It is not difficult to assess the impact of the NP meta-grid on the overall device performance, however, we have not focused on calculating that. Typically, an encapsulant layer or the epoxy cap of an LED chip is made in hemispherical shape. This ensures that emitted light is incident on epoxy/air interface at almost normal incidence, so that there is no critical angle loss. However, there is some Fresnel loss across that epoxy/air interface, which is marginal due to not-so-large difference in the refractive indices of epoxy and air. For an epoxy (*n* = 1.58)/air (*n* = 1) interface, the Fresnel reflection loss accounts for 5.05% of intensity at normal incidence. This means 94.95% light impinging on that interface is transmitted out. Our NP meta-grid enhances the amount of light impinging on this interface by allowing an increase in light transmission from emissive layer to encapsulant (epoxy) layer to ~96% (within the photon escape cone), which is otherwise ~84%. Thus, one can readily estimate how much impact our NP meta-grid has on the overall device performance, which will be almost solely based on the transmission enhancement through the chip/epoxy interface after the insertion of the optimized NP meta-grid. One may put another detector at the outermost medium to get an accurate value or do some quick back-of-the-envelope calculation for an adequate estimate. A quick, back-of-the-envelope calculation shows that with NP meta-grid the overall device performance would increase by a factor of ~1.15 (for the spectral range considered and within the photon escape cone), with increase by a factor of ~1.18 at the peak wavelength of the considered LED at normal incidence. These estimates are made from the ratio of intensities of light transmitted to air from the emissive layer with and without the optimized NP meta-grid, given by $$\begin{array}{l}(I \,\times\, T_{{\mathrm{with}}\,{\mathrm{NPs}}} \,\times\, T_{{\mathrm{epoxy/air}}\,{\mathrm{interface}}})/(I \,\times\, T_{{\mathrm{without}}\,{\mathrm{NPs}}} \,\times\, T_{{\mathrm{epoxy/air}}\,{\mathrm{interface}}})\\ \,\cong\, T_{{\mathrm{with}}\,{\mathrm{NPs}}}/T_{{\mathrm{without}}\,{\mathrm{NPs}}}\end{array}$$, where *I* is the intensity of the light emitted by the emissive layer.

## Materials and methods

The theoretical framework, based on a four-layer-stack model (see Fig. 1d), combines effective medium theory with multilayer Fresnel reflection scheme^27^. Here, the optical response of the monolayer of NPs is modeled (within quasi-static dipolar approximation) as an effective film, the anisotropic dielectric permittivity of which is determined by the optical polarizability of the NPs, their interaction with the neighboring media and the structure of the NP array.

The relative permittivity of layer 1, representing the semiconductor material of the LED structure, is denoted by *ε*_1_, whereas those of layers 2 and 4 are represented by relative permittivities *ε*_2_ and *ε*_4_, respectively. Both layers, 2 and 4, comprise the same encapsulating medium of the LED, which implies that *ε*_2_ = *ε*_4_. In this approach, the critical step is to determine the frequency-dependent permittivity *ε*_3_(*ω*) and thickness *d* of layer 3 embedded in a medium of permittivity *ε*_2_.

A monolayer of identically-sized, spherical NPs formed by a self-assembly process, typically arranges in a hexagonal lattice, which can be characterized by lattice constant *a* (=2*R* + *g*, where *R* is the radius and *g* is the gap between NPs). The effective film, of thickness $d \,=\, \frac{{4\pi R^3}}{{3a^2}}$, which represents such an NP array, has an anisotropic optical response, with different parallel and perpendicular components of the dielectric tensor denoted as $\varepsilon _3^\parallel \left( \omega \right)$and $\varepsilon _3^ \bot \left( \omega \right)$, respectively:^27,30,35^

1a $$\varepsilon _3^\parallel \left( \omega \right) \,=\, \varepsilon _2 \,+\, \frac{{8\pi }}{{\surd 3a^2d}}\beta ^\parallel (\omega )$$

1b $$\frac{1}{{\varepsilon _3^ \bot (\omega )}} \,=\, \frac{1}{{\varepsilon _2}} \,-\, \frac{1}{{\varepsilon _2^2}}\frac{{8\pi }}{{\surd 3a^2d}}\beta ^ \bot (\omega )$$

where $\beta ^{\parallel , \bot }(\omega )$ is the effective quasi-static dipolar polarizability of each NP—interacting with all the other NPs of the array along with their dipolar images—

2a $$\beta ^\parallel (\omega ) \,=\, \frac{{\alpha (\omega )}}{{1 \,+\, \alpha \left( \omega \right)\frac{1}{{\varepsilon _2}}\left[ {\frac{{ - 1}}{2}\frac{{U_A}}{{a^3}} \,-\, \xi \left( {\frac{{f(h,a)}}{{a^3}} \,-\, \frac{3}{2}\frac{{g_1\left( {h,a} \right)}}{{a^3}} \,+\, \frac{1}{{8h^3}}} \right)} \right]}}$$

2b $$\beta ^ \bot (\omega ) \,=\, \frac{{\alpha (\omega )}}{{1 \,+\, \alpha \left( \omega \right)\frac{1}{{\varepsilon _2}}\left[ {\frac{{U_A}}{{a^3}} \,-\, \xi \left( {\frac{{f(h,a)}}{{a^3}} \,-\, 12\frac{{h^2g_2\left( {h,a} \right)}}{{a^5}} \,-\, \frac{1}{{4h^3}}} \right)} \right]}}$$

here $\alpha (\omega ) \,=\, \varepsilon _2R^3\frac{{\varepsilon _{{\mathrm{NP}}}(\omega ) \,-\, \varepsilon _2}}{{\varepsilon _{{\mathrm{NP}}}(\omega ) \,+\, 2\varepsilon _2}}$ is the quasi-static dipolar polarizability of a lone sub-wavelength spherical NP made of a material with permittivity *ε*_NP_(*ω*), embedded in a medium of permittivity *ε*_2_. The image-charge screening factor is $\xi \,=\, \frac{{\varepsilon _2 \,-\, \varepsilon _1}}{{\varepsilon _2 \,+\, \varepsilon _1}}$; it can be *ω*-dependent if either or both *ε*_1_ and *ε*_2_ depend on the frequency of light. The lattice parameters—*U*_*A*_, *f* (*h, a*), *g*_1_ (*h, a*), and *g*_2_ (*h, a*) are calculated from the following summations over the hexagonal lattice^27,35^:

3a $$U_A \,=\, \mathop {\sum}\limits_i^\prime {\mathop {\sum}\limits_j^\prime {\frac{1}{{\left( {i^2 \,+\, j^2 \,-\, ij} \right)^{3/2}}} \,=\, 11.031} }$$

3b $$f(h,a) \,=\, \mathop {\sum}\limits_i^\prime {\mathop {\sum}\limits_j^\prime {\frac{1}{{\left( {i^2 \,+\, j^2 \,-\, ij \,+\, \left( {\frac{{2h^\prime }}{a}} \right)^2} \right)^{3/2}}}} }$$

3c $$g_1(h,a) \,=\, \mathop {\sum}\limits_i^\prime {\mathop {\sum}\limits_j^\prime {\frac{{\left( {i^2 \,+\, j^2} \right)}}{{\left( {i^2 \,+\, j^2 \,-\, ij \,+\, \left( {\frac{{2h^\prime }}{a}} \right)^2} \right)^{5/2}}}} }$$

3d $$g_2(h,a) \,=\, \mathop {\sum}\limits_i^\prime {\mathop {\sum}\limits_j^\prime {\frac{1}{{\left( {i^2 \,+\, j^2 \,-\, ij \,+\, \left( {\frac{{2h^\prime }}{a}} \right)^2} \right)^{5/2}}}} }$$

where $h^\prime \,=\, h \,+\, R$ with *h* denoting the separation of the NP array from layer 1 (see Fig. 1).

Once the permittivity values for all the layers are known, a multilayer Fresnel reflection scheme can be deployed to calculate the transmission through the entire system under study. The detailed calculation for obtaining the transmission coefficient *t*^(s,p)^ from a four-layer-stack model, for both s- and p-polarizations of incident light, is presented in ref. ^27^. Note that in the LED device, the light generated in the high refractive index material of the LED chip is intended to be extracted into the low refractive index LED-encapsulating medium, a scenario inverse to the one described in ref. ^27^.

The transmittance *T*^(s,p)^ from the four-layer-stack system can be calculated as

4 $$T^{({\mathrm{s}},{\mathrm{p}})} \,=\, \left| {t^{({\mathrm{s}},{\mathrm{p}})}} \right|^2 \,\times\, \frac{{n_4}}{{n_1}}\frac{{\cos \theta _t}}{{\cos \theta _i}}$$

where $\theta _t \,=\, \sin ^{ - 1}\left( {\frac{{n_{1\sin \theta _i}}}{{n_4}}} \right)$ is the angle of transmission, *θ*_*i*_ is the angle of incidence, *n*_1_ and *n*_4_ (=*n*_2_) are the refractive indices of the LED’s semiconductor and the encapsulating materials, respectively. In Eq. (4), the transmittance is calculated as a fraction of unity. In all figures, throughout the paper, we present transmission in percentage, after multiplying the results by 100.

The theoretically calculated transmittance spectra were verified using full-wave simulations. These were conducted using the “RF module”—an add-on to the commercial FEM software COMSOL Multiphysics^®^. RF module allows designing and analyzing devices working in radio frequency, microwave, mm wave, THz, as well as visible spectral regime in various scenarios. The simulation setup was developed mainly based on the four-layer-stack model shown in Fig. 1d, replacing the NP-equivalent film layer of the effective medium theory^27^ by an actual NP meta-grid of silver nanospheres. The structure of the grid was assumed to be a 2D hexagonal array, which is the usual preferred packing order of self-assembled spherical NPs. A unit cell of the hexagonal array was modeled first and then extended in both lateral dimensions using periodic boundary conditions to emulate the NP meta-grid. For capturing the fine structural details of NP meta-grid, we considered extremely fine meshing, which has a minimum element size of *λ*/10 (with *λ* being the wavelength of light in the medium in which the grid is embedded). Source and destination ports were deployed at the emitter and the detector ends, respectively. Using the frequency domain solver over the desired spectral range, the transmittance spectrum was obtained from *S*_21_ coefficient of the numerically calculated *S* parameters^31^.
